# GLP-1 Analogue Liraglutide Enhances SP-A Expression in LPS-Induced Acute Lung Injury through the TTF-1 Signaling Pathway

**DOI:** 10.1155/2018/3601454

**Published:** 2018-05-22

**Authors:** Tao Zhu, Changyi Li, Xue Zhang, Chunyan Ye, Shuo Tang, Wei Zhang, Jiayang Sun, Niwen Huang, Fuqiang Wen, Daoxin Wang, Huojin Deng, Jing He, Di Qi, Wang Deng, Tao Yang

**Affiliations:** ^1^Division of Pulmonary Diseases, State Key Laboratory of Biotherapy of China, and Department of Respiratory Medicine, West China Hospital of Sichuan University, Chengdu 610041, China; ^2^Respiratory Medicine, Second Affiliated Hospital of Chongqing Medical University, Chongqing 400010, China; ^3^Luoyang Orthopedic Hospital of Henan Province, Luoyang 471000, China; ^4^School Hospital of Southern Medical University, Guangzhou 510280, China; ^5^Pain Medicine, Shenzhen Nanshan Hospital, Shenzhen 518052, China; ^6^Respiratory Medicine, First Affiliated Hospital of Chengdu Medical College, Chengdu, Sichuan 610500, China; ^7^Respiratory Medicine, Affiliated Hospital of Guiyang Medical University, Guiyang 550004, China; ^8^Respiratory Medicine, Zhujiang Hospital of Southern Medical University, Guangzhou 510280, China; ^9^Thoracic Surgery, First Affiliated Hospital of Chongqing Medical University, Chongqing 400016, China

## Abstract

The reduction of pulmonary surfactant (PS) is essential for decreased pulmonary compliance and edema in acute lung injury (ALI). Thyroid transcription factor-1 (TTF-1) plays a major role in the regulation of surfactant protein-A (SP-A), the most abundant protein component of PS. Simultaneously, the glucagon-like peptide-1 (GLP-1) analogue can enhance SP-A expression in the lung. However, the underlying mechanism is still unknown. The purpose of this study was to explore whether liraglutide, a GLP-1 analogue, upregulates SP-A expression through the TTF-1 signaling pathway in ALI. In vivo, a murine model of ALI was induced by lipopolysaccharide (LPS). Pulmonary inflammation, edema, insulin level, ultrastructural changes in type II alveolar epithelial (ATII) cells, and SP-A and TTF-1 expression were analyzed. In vitro, rat ATII cells were obtained. SP-A and TTF-1 expression in cells was measured. ShRNA-TTF-1 transfection was performed to knock down TTF-1 expression. Our data showed that LPS-induced lung injury and increase in insulin level, and LPS-induced reduction of SP-A and TTF-1 expression in both the lung and cells, were significantly compromised by liraglutide. Furthermore, we also found that these effects of liraglutide were markedly blunted by shRNA-TTF-1. Taken together, our findings suggest that liraglutide enhances SP-A expression in ATII cells and attenuates pulmonary inflammation in LPS-induced ALI, most likely through the TTF-1 signaling pathway.

## 1. Introduction

Pulmonary surfactant (PS), a complex of various lipids and proteins lining the alveolar surface, is synthesized by type II alveolar epithelial (ATII) cells [1, 2]. Its principle function is to maintain pulmonary compliance and pulmonary fluid balance, to prevent the lung from collapsing at the end of expiration, and to regulate the size of alveoli [1, 2]. Meanwhile, PS also plays a role in pulmonary immune defenses [3].

Acute lung injury (ALI)/acute respiratory distress syndrome (ARDS) is one of most common fatal diseases with an extremely high morbidity rate in critically ill patients [4]. Alveolar epithelial and endothelial cells are the major injury targets of ALI/ARDS [5, 6]. It is well known that reduction of PS is essential for decreased pulmonary compliance and pulmonary edema in ALI/ARDS [7, 8]. Surfactant protein-A (SP-A), a hydrophilic collectin, is the most abundant protein component of PS [9]. The main function of SP-A is to facilitate tubular myelin formation; however, it also plays an active role in defense against pathogens and immunological regulation in the lung [3, 9–11]. Simultaneously, several studies have shown that thyroid transcription factor-1 (TTF-1), also known as Nkx2.1, contributed substantially to the regulation of SP-A expression in ATII cells [12–14]. Chen et al. found that hypoxia-induced reduction of SP-A expression was attenuated by leptin through promoting TTF-1 translation in rat ATII cells [13].

Glucagon like peptide-1 (GLP-1), a peptide hormone synthesized and secreted by the L-cells in guts, is essential for glycometabolism and lipid metabolism. Recently, the GLP-1 receptor (GLP-1R), a transmembrane G-protein coupled receptor, has been found in a variety of tissues outside of the pancreas, such as the lung, heart, blood vessels, and kidney [15–17]. Viby et al. showed that GLP-1R was wildly expressed in the airway and alveolar epithelium, in both mice and humans [16]. Romaní-Pérez et al. reported that liraglutide, a GLP-1 analogue, upregulated SP-A expression in the fetal lung and promoted lung development in newborn rats [17]. However, the underlying mechanism remains unclear. In addition, some studies also confirmed that GLP-1 possessed a potent anti-inflammatory property in different inflammatory conditions [15, 18–20]. Our recent study also showed that ovalbumin- (OVA-) induced airway inflammation and mucus hypersecretion were significantly suppressed by liraglutide in a murine model of asthma [15].

Therefore, the purpose of the current study was to explore whether GLP-1 analogue liraglutide upregulates SP-A expression in ATII cells and attenuates inflammation in LPS-induced ALI and to elucidate its underlying mechanism.

## 2. Methods and Materials

### 2.1. Animals

All procedures involving animals were approved by the Animal Experimental Ethics Committee of West China Medical School of Sichuan University. The current study was performed according to the recommendations in the Guide for the Care and Use of Laboratory Animals. All surgeries were performed using sodium pentobarbital anesthesia, and all efforts were made to minimize suffering. Six–eight-week-old male BALB/c mice (18–22 g) and male Sprague-Dawley (SD) rats (200–220 g) were maintained under specific pathogen-free conditions in the animal center facilities of our University. The mice and rats were kept in a temperature-controlled room (12 h dark/light cycles) and offered ad libitum access to food and water. Animals underwent an acclimatization period of at least 1 week before study.

### 2.2. Knockdown of TTF-1 by shRNA in Mice

To silence the expression of TTF-1 in the lung, a recombinant lentiviral vector for TTF-1 (shRNA-TTF-1, sc-36757-V, Santa Cruz Biotechnology, CA, USA) was used. Lentivirus-expressing nontargeting sequences (sc-108080; Santa Cruz Biotechnology, CA, USA) were used as the negative control (shRNA-scramble). In brief, thirty male BALB/c mice were divided into 3 groups—control, shRNA-scramble, and shRNA-TTF-1 groups—with 10 mice in each group. After anesthesia, shRNA-TTF-1 lentiviral vector (40 *μ*L) for the shRNA-TTF-1 group or negative control lentivirus (40 *μ*L) for the shRNA-scramble group was given by intratracheal injection. The mice in the control group were then administered sterile saline. Three days after transfection, the left lower lung was resected. Histology changes were observed by HE staining. The efficiency of shRNA transfection was measured by qPCR and Western blot analysis.

### 2.3. Murine Model of LPS-Induced ALI

Fifty male BALB/c mice were randomly and evenly divided into 5 groups: control, liraglutide (Lira), LPS, LPS+Lira, and LPS+Lira+shRNA-TTF-1 groups, with 10 mice in each group. ALI was induced by intratracheal injection of LPS (*Escherichia coli*; serotype 0111:B4; Sigma-Aldrich, St. Louis, MO, USA) [6, 21]. In brief, mice were anesthetized with 30 mg/kg of pentobarbital sodium, followed by intratracheal injection of 10 *μ*g of LPS in 50 *μ*L sterile saline with a 3-gauge needle. ShRNA-TTF-1 (40 *μ*L) was also given by intratracheal injection 3 days before LPS stimulation. The mice in the control group were administrated sterile saline instead. According to our previous studies, liraglutide (2 mg/kg in 200 *μ*L sterile saline, Novo Nordisk A/S, Novo Alle, DK-2880 Bagsvaerd, Denmark) was given by intraperitoneal injection, 20 min after LPS injection [15, 22]. In 3 days, liraglutide was given every 12 h (7 times in total).

### 2.4. Bronchoalveolar Lavage Fluid (BALF) and Cell Counting

Seventy-two hours later, mice were sacrificed under anesthesia by pentobarbital (50 mg/kg i.p.). BALF was collected by cannulating the upper part of the trachea, by lavage 3 times with 1.0 mL phosphate-buffered saline (pH 7.2) [23]. The fluid recovery rate was about 90%. The sediment cells were stained with Diff-Quik (International Reagents Corp., Japan) for cytospin preparations. The number of total cells, neutrophils, macrophages, and lymphocytes was then counted double-blindly with a hemocytometer.

### 2.5. TNF-*α*, IL-6, and IL-1*β* in BALF

As described before, the BALF supernatant was collected and stored at −80°C before performing the cytokine assay [6, 21]. TNF-*α*, IL-6, and IL-1*β* expression levels in BALF were measured by ELISA (R&D Systems, USA).

### 2.6. Myeloperoxidase (MPO) Activity Assay

Seventy-two hours after LPS injection, mice were sacrificed and their lungs were collected. According to our previous studies, MPO activity was detected. Results are expressed as units of MPO activity per gram of lung tissue [6, 21].

### 2.7. Lung Wet/Dry Weight Ratio

Seventy-two hours after LPS injection, mice were sacrificed and their lungs were collected. The severity of pulmonary edema was assessed by the wet-to-dry ratio (W/D) [6, 21]. The left lower lung was weighed and then dehydrated at 60°C for 72 h in an oven.

### 2.8. H&E Staining and Lung Injury Score

Seventy-two hours after LPS injection, mice were sacrificed and their lungs were collected. The right lower lung of each mouse was fixed in 10% formalin, embedded in paraffin, cut into 5 *μ*m sections, and stained with H&E to observe the pathological alterations in the lung tissues. According to our previous studies, the lung injury score was measured by a blinded pathologist with a 0- to 4-point scale according to combined assessments of inflammatory cell infiltration in the airspace or vessel wall, alveolar congestion, hemorrhage, alveolar wall thickness, and hyaline membrane formation [6, 21]. Five microscope fields from each histological section were taken and scored. The final score of each sample was the average of 5 scores. Briefly, a score of 0 represented no damage, 1 represented mild damage, 2 represented moderate damage, 3 represented severe damage, and 4 represented very severe histological changes.

### 2.9. Immunohistochemistry

Seventy-two hours after LPS injection, mice were sacrificed and their lungs were collected. SP-A expression in the lung was determined by immunohistochemistry. Briefly, tissue sections (right lower lung) were deparaffinated and rehydrated. Samples were treated with Target Retrieval (Dako, Glostrup, Denmark) at 95°C, blocked at room temperature with Protein Block Serum-Free (Dako, Glostrup, Denmark), and incubated with anti-SP-A antibody (1 : 400; sc-7699, Santa Cruz Biotechnology, Santa Cruz, CA, USA). After washing, sections were incubated with biotin-conjugated anti-rabbit immunoglobulin G (IgG) for 30 min at room temperature. The biotinylated reagents were detected with ABC complex HRP (Dako, Glostrup, Denmark).

### 2.10. Transmission Electron Microscopy (TEM)

Seventy-two hours after LPS injection, mice were sacrificed and their lungs were collected. TEM was performed to observe the ultrastructural changes in pulmonary epithelial cells and to identify rat ATII cells [21, 24]. Lamellar bodies, the principal storage site of pulmonary surfactant, are the specific organelle and the feature of ATII cells. In brief, the fresh left upper lung tissues and isolated cells were obtained for observation. Images were taken with an electron microscope (H-600IV, Hitachi, Tokyo, Japan).

### 2.11. Fasting Serum Insulin

Blood was taken from the tail vein without anesthesia, 72 h after LPS injection and 6 h after the last feeding, before sacrifice. The procedure was done in the afternoon. According to the instructions of the manufacturer, the fasting serum insulin level was analyzed using an ELISA kit (ALPCO Diagnostics).

### 2.12. Rat ATII Cell Isolation and Characterization

Rat ATII cells were isolated from male pathogen-free SD rats (200–220 g) as described previously [25]. In brief, the lungs were perfused via the pulmonary artery to remove the blood. The lung was digested with intratracheally instilled 3 U/mL elastase (Sangon Biotech, Shanghai, China) three times at 37°C for 40 minutes. Due to Fc*γ* receptors (Fc*γ*R), the receptor of IgG, only expressed on non-ATII cells in the lung, rat IgG can be used to remove non-ATII cells in the lung. In our study, ATII cells were purified by differential adhesion to IgG-pretreated dishes (Boster Biological Technology, Wuhan, China). More than 95% of the cells obtained were viable, which was assessed by trypan blue exclusion assay. Cells were resuspended in DMEM supplemented with FBS (10%), penicillin (100 U/mL), and streptomycin (100 *μ*g/mL). Cells were then used in experiments after a 24 h culture period. TEM was used to identify the ATII cells, as mentioned above.

### 2.13. Cell Transfection with shRNA

According to our previous study, ATII cells were transfected at 70% confluence with shRNA-TTF-1 (336312, Qiagen, Valencia, CA) or shRNA-scrambled (sc-108060, Santa Cruz Biotechnology, CA, USA) [26]. Twenty-four hours after transfection, ATII cells were used for further experiments. Meanwhile, total and nuclear proteins and mRNA were extracted from cells and kept at −80°C for qPCR and Western blot. Then, knockdown of TTF-1 expression was analyzed by qPCR and Western blot. *β*-Actin was used as an internal control.

### 2.14. Cell Intervention

ShRNA-TTF-1-transfected and nontransfected ATII cells were pretreated with liraglutide (100 nM) for 4 h and then stimulated with LPS (100 ng/mL) for 4 h [21, 26, 27]. Then, total and nuclear proteins and mRNA were extracted from cells and kept at −80°C for qPCR and Western blot. MTT assay (Promega, Madison, WI) was used to assess cell viability at 0 h and 4 h after interventions.

### 2.15. Quantitative PCR

The mRNA expression of SP-A and TTF-1 was detected by qPCR [15, 22]. *β*-Actin was used as an internal reference. Briefly, mice were sacrificed and their lungs were collected. The right upper lung tissues were kept at −80°C. Then, total RNA of lung tissues and rat ATII cells were isolated by TRIzol reagent. PrimeScript® RT reagent kit with gDNA eraser (Takara Bio Inc., Otsu, Japan) was used for reverse transcription. PCR was then performed with iQ™5 Multicolor Real-Time PCR Detection System (Bio-Rad Laboratories Inc., USA) and a SYBR Green PCR kit (Takara Bio Inc., Otsu, Japan) in a final volume of 20 *μ*L, containing 1.6 *μ*L cDNA template, forward and backward primers (0.8 *μ*L each), 10 *μ*L SYBR® Premix Ex Taq™ II, and 6.8 *μ*L dH_2_O. The primers and TaqMan probes were designed using Primer Premier (PREMIER Biosoft International, Canada). The premier sequences were as follows. mTTF-1: (forward) 5′-AACAGC GGCCATGCAG CAGCAC-3′ and (reverse) 5′-CCATG TTCTTGC TCACGTCC-3′; mSP-A: (forward) 5′-TCGGA GGCAGACA TCCACA-3′ and (reverse) 5′-GCCAGCA ACAACAGTC AAGAAGAG-3′; m*β*-actin: (forward) 5′-GATTA CTGCTCTGG CTCCTAGC-3′ and (reverse) 5′-ACTCAT CGTACTCC TGCTTGCT-3′; rTTF-1: (forward) 5′-AAATT TGGGGGT CTTTCTGG-3′ and (reverse) 5′-AGAGT GCATCCA CAGGGAAG-3′; rSP-A: (forward) 5′-AGCCTG CAGGTCTG TATGTGGA-3′ and (reverse) 5′-TTGCAC TTGATACCA GCGACAAC-3′; and r*β*-actin: (forward) 5′-ATCATGTT TGAGACCT TCAACA-3′ and (reverse) 5′-CATCTC TTGCTCGA AGTCCA-3′. Changes in the expression of target genes were calculated using the 2^−△△Ct^ method, △△Ct = (Ct_target_ − Ct_*β*−actin_)_sample_ − (Ct_target_ − Ct_*β*−actin_)_control_.

### 2.16. Western Blot

Western blot was performed to evaluate the protein expression [21, 28]. Briefly, 72 hours after LPS injection, mice were sacrificed and their lungs were collected. The lung tissues were kept at −80°C. Protein lysates from the left upper lung tissues and rat ATII cells were subjected to sodium dodecyl sulfate-polyacrylamide gel electrophoresis and then transferred to nitrocellulose membranes. Antibodies against mSP-A, mTTF-1, rSP-A, rTTF-1, m*β*-actin, and r*β*-actin were purchased from Santa Cruz Biotechnology (Santa Cruz, CA, USA). The relative protein levels of mSP-A, rSP-A, mTTF-1, and rTTF-1 were normalized to that of *β*-actin.

### 2.17. Statistical Analysis

Statistical analyses were performed with SPSS software, version 17.0 (SPSS Inc., Chicago, IL, USA). All data were presented as mean ± standard error of mean (SEM). One-way analysis of variance (ANOVA) with Student-Newman-Keuls (SNK) test was performed. *P* < 0.05 was considered to be statistically significant.

## 3. Results

### 3.1. TTF-1 Expression Was Inhibited after Transfection of the Lung with shRNA-TTF-1

**Figure 1:**
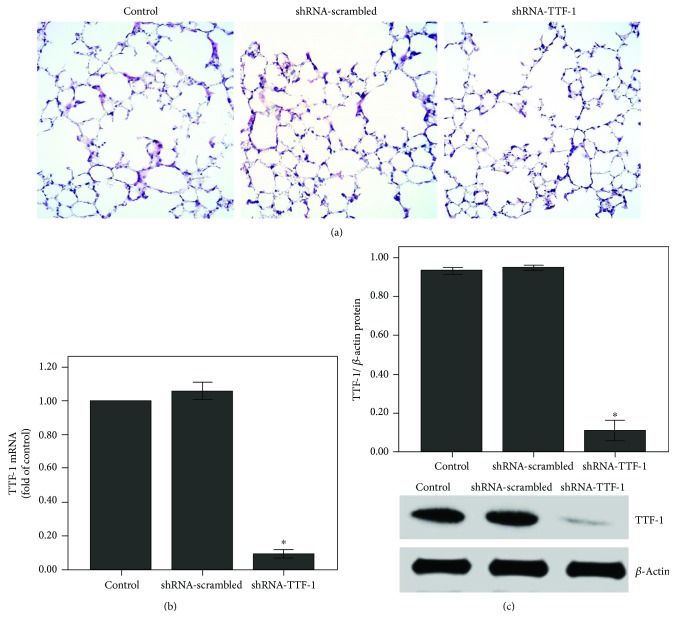
The expression of TTF-1 was inhibited after shRNA-TTF-1 transfection in the lung. Mice were transfected with shRNA-TTF-1 or shRNA-scrambled by intratracheal injection. Seventy-two hours after transfection, the expression of TTF-1 was measured. (a) After 72 h of transfection, mice were sacrificed and their right lower lungs were fixed. The tissue sections were then stained with H&E. The figure demonstrates a representative view (×200) from each group. (b) QPCR was used to analyze the mRNA expression of TTF-1. (c) Western blot was performed to evaluate the protein expression of TTF-1. Each bar represents the mean ± SEM of 10 mice. ^∗^*P* < 0.05 compared with control.

After 3 days of transfection, no pathological alterations were observed in the control, shRNA-scrambled, shRNA-TTF-1 groups (Figure 1(a)). However, TTF-1 expression was largely inhibited by shRNA-TTF-1 in the lung (Figures 1(b) and 1(c)).

### 3.2. Liraglutide Attenuated Pulmonary Inflammation and Pulmonary Edema in LPS-Induced ALI

**Figure 2:**
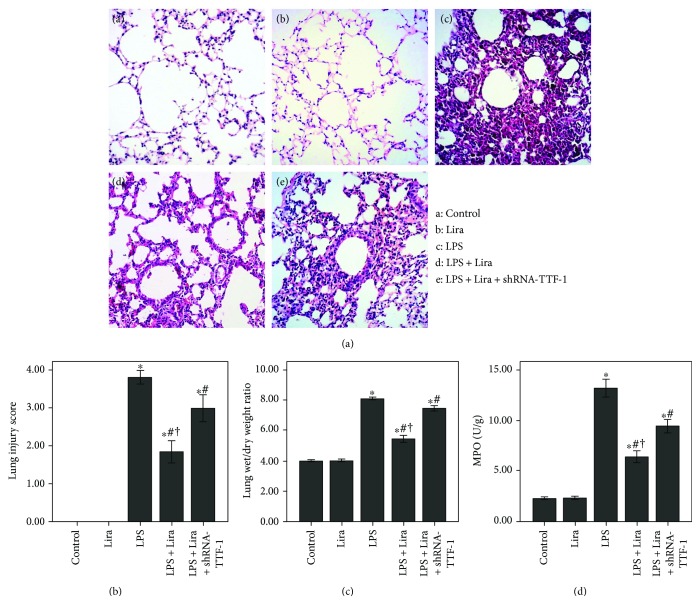
Liraglutide attenuated pulmonary inflammation and pulmonary edema in LPS-induced ALI. (a) After 72 h of intervention, mice were sacrificed and their right lower lungs were fixed. The tissue sections were then stained with H&E. The figure demonstrates a representative view (×200) from each group. (b) Severity of lung injury was measured by the lung injury scoring system. (c) The left lower lungs were obtained to evaluate the W/D ratio of the lung tissues. (d) MPO activity was measured to evaluate the accumulation and activation of neutrophils in the lung tissues. Each bar represents the mean ± SEM of 10 mice. ^∗^*P* < 0.05 compared with control. ^#^*P* < 0.05 compared with LPS. ^†^*P* < 0.05 compared with LPS+Lira+shRNA-TTF-1.

After 72 h of LPS injection, severe and typical pulmonary pathological alterations were observed, including severe and extensive inflammatory cell infiltration, interstitial and intra-alveolar edema and patchy hemorrhage, interalveolar septal thickening, and hyaline membrane formation with some alveoli collapsed (Figure 2(a)). As shown in Figure 2(c), the W/D ratio was significantly increased after 72 h of LPS injection.  Figure 2(d) demonstrates that MPO activity was significantly enhanced by LPS injection. However, LPS-induced lung injury, W/D ratio, and MPO activity were all notably suppressed by liraglutide (Figure 2). Furthermore, these effects of liraglutide were substantially blunted by shRNA-TTF-1. No pathological changes were observed in the control and Lira groups (Figure 2(a)).

### 3.3. Liraglutide Reduced Inflammatory Cell Counts and Inflammatory Mediators in BALF

**Figure 3:**
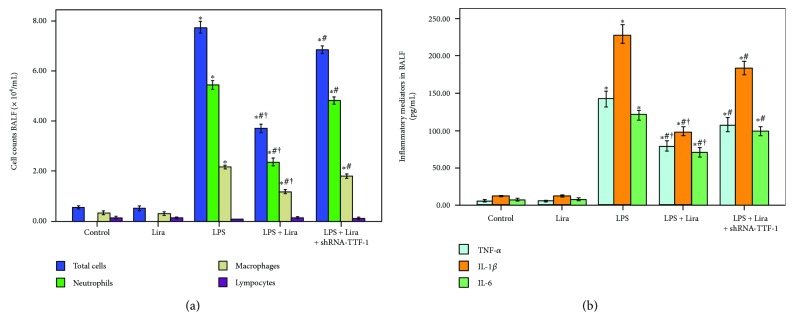
Liraglutide reduced inflammatory cell counts and inflammatory mediators in BALF. (a) Cells in BALF were collected, and cytospin preparations were made. The number of total cells, neutrophils, macrophages, and lymphocytes in BALF were assessed. (b) TNF-*α*, IL-6, and IL-1*β* levels in BALF were detected by ELISA. Each bar represents the mean ± SEM of 10 mice. ^∗^*P* < 0.05 compared with control. ^#^*P* < 0.05 compared with LPS. ^†^*P* < 0.05 compared with LPS+Lira+shRNA-TTF-1.

As shown in Figure 3, the number of total cells, neutrophils, and macrophages and the levels of TNF-*α*, IL-6, and IL-1*β* in BALF were notably increased after 72 h of LPS injection. Meanwhile, our data also demonstrated that the increase in the number of total cells, neutrophils, and macrophages and increased levels of TNF-*α*, IL-6, and IL-1*β* in BALF, induced by LPS, were remarkably reduced by liraglutide. These effects of liraglutide were substantially blocked by shRNA-TTF-1. No difference in the number of lymphocytes in BALF was found among all groups (Figure 3(a)).

### 3.4. Liraglutide Alleviated Pathological Alterations in ATII Cell Ultrastructure in LPS-Induced ALI

**Figure 4:**
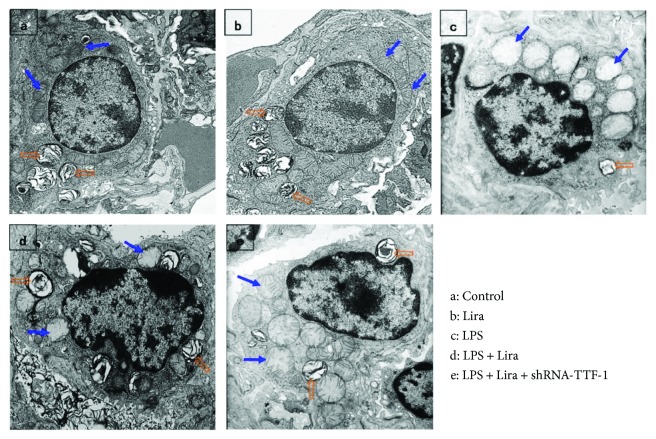
Liraglutide alleviated pathological alterations in ATII cell ultrastructure in LPS-induced ALI. After mice were sacrificed, the left upper lung tissues were taken and observed by TEM. The figure demonstrates a representative view (×6000) from each group. Mitochondrion, blue arrow; lamellar body, orange open arrow.

After 72 h of LPS injection, significant ultrastructural pathological alterations were observed in ATII cells. These features included cell swelling with a cytoplasm of low electronic density, unclear cell structure, mitochondrial edema with dilated mitochondrial cristae, chromatin margination, and reduced and indistinct cell surface microvilli, along with decreased and vacuolated lamellar bodies (Figure 4). Meanwhile, as shown in Figure 4, these ultrastructural alterations were alleviated by liraglutide. However, shRNA-TTF-1 notably abolished this effect of liraglutide. No ultrastructural pathological change was observed in the control and Lira groups.

### 3.5. Liraglutide Enhanced SP-A Expression by Increasing TTF-1 in LPS-Induced ALI

**Figure 5:**
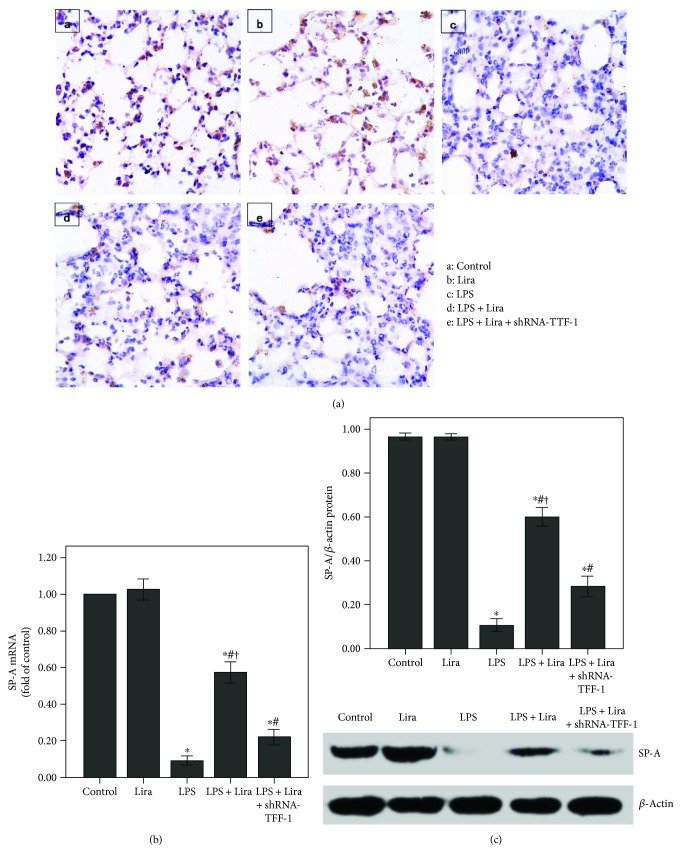
Liraglutide enhanced SP-A expression in LPS-induced ALI. (a) After 72 h of interventions, mice were sacrificed and their right lower lungs were fixed. Immunohistochemistry was then performed to observe SP-A expression in the lung. (b) QPCR was used to analyze the mRNA expression of SP-A. (c) Western blot was performed to evaluate the protein expression of SP-A. Each bar represents the mean ± SEM of 10 mice. ^∗^*P* < 0.05 compared with control. ^#^*P* < 0.05 compared with LPS. ^†^*P* < 0.05 compared with LPS+ Lira+shRNA-TTF-1.

**Figure 6:**
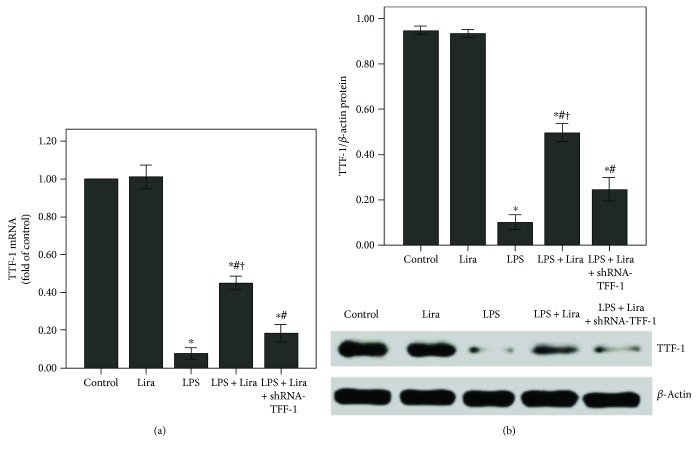
Liraglutide increased the expression of TTF-1 in LPS-induced ALI. (a) QPCR was used to analyze the mRNA expression of TTF-1. (b) Western blot was performed to evaluate the protein expression of TTF-1. Each bar represents the mean ± SEM of 10 mice. ^∗^*P* < 0.05 compared with control. ^#^*P* < 0.05 compared with LPS. ^†^*P* < 0.05 compared with LPS+Lira+shRNA-TTF-1.

As shown in Figures 5(b) and 5(c), the expression of SP-A was substantially decreased after 72 h of LPS injection, and liraglutide significantly enhanced SP-A expression in the lung after LPS injection. In addition, SP-A expression in the ATII cells was reduced after LPS administration (Figure 5(a)). Subsequently, as shown in Figure 6, LPS-induced suppression of TTF-1 expression was markedly alleviated by liraglutide. Furthermore, our data also demonstrated that these effects of liraglutide were largely abrogated by shRNA-TTF-1.

### 3.6. Liraglutide Reduced Serum Insulin Level in LPS-Induced ALI

**Figure 7:**
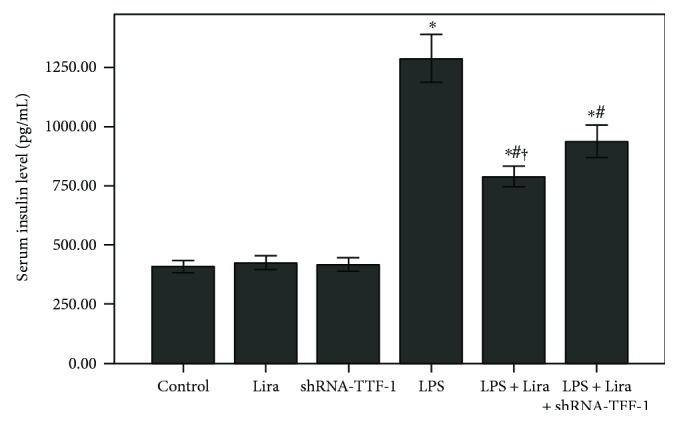
Liraglutide reduced the serum insulin level in LPS-induced ALI. Fasting serum insulin level (72 h after LPS injection and 6 h after last feeding) was detected by ELISA. Each bar represents the mean ± SEM of 10 mice. ^∗^*P* < 0.05 compared with control. ^#^*P* < 0.05 compared with LPS. ^†^*P* < 0.05 compared with LPS+Lira+shRNA-TTF-1.

Fasting serum insulin level (72 h after LPS injection, 6 h after last feeding) was measured. Fasting serum insulin level was markedly increased after 72 h of LPS injection. Meanwhile, as shown in Figure 7, the increased level of fasting serum insulin induced by LPS was significantly reduced by liraglutide. This effect of liraglutide was substantially blocked by shRNA-TTF-1. No difference in the level of serum insulin was found among the control, Lira, and shRNA-TTF-1 groups.

### 3.7. Identification of Rat ATII Cells and Evaluation of Cell Viability

**Figure 8:**
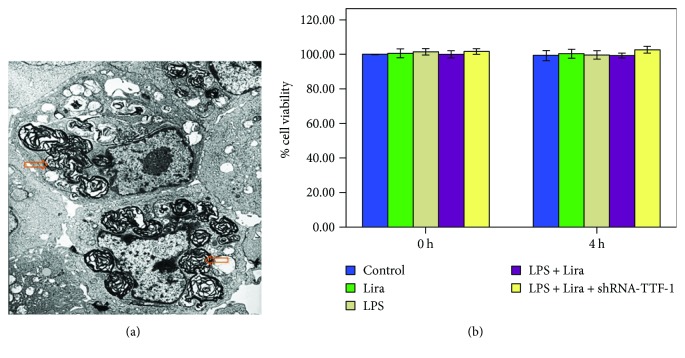
Identification of rat ATII cells and evaluation of cell viability. (a) Isolated and cultivated rat ATII cells were identified by TEM. The figure demonstrates a representative view (×6000). Lamellar body, orange open arrow. (b) MTT assay was performed to evaluate rat ATII cell viabilities. Quantitative data were presented as mean ± SEM (*n* = 5). ^∗^*P* < 0.05 compared with control.

Isolation primary rat ATII cells were confirmed by TEM. Lamellar bodies, the characteristic organelles of ATII cells, were observed in our isolated cells (Figure 8(a)). To evaluate the cell viability of rat ATII cells, MTT assay was performed. As shown in Figure 8(b), no significant difference in cell viability was found between different groups after 4 h of intervention.

### 3.8. The Expression of TTF-1 Was Inhibited after shRNA-TTF-1 Transfection in Rat ATII Cells

**Figure 9:**
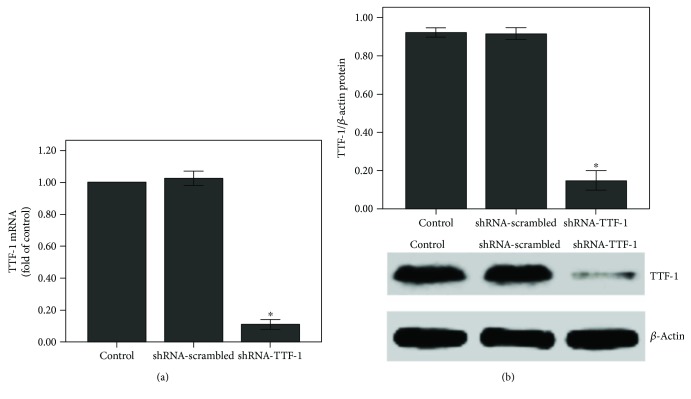
The expression of TTF-1 was inhibited after shRNA-TTF-1 transfection in rat ATII cells. Rat ATII cells were transfected with shRNA-TTF-1 or shRNA-scrambled. Twenty-four hours after transfection, the expression of TTF-1 was measured. (a) QPCR was used to analyze the mRNA expression of TTF-1. (b) Western blot was performed to analyze the protein expression of TTF-1. Quantitative data were presented as mean ± SEM (*n* = 5). ^∗^*P* < 0.05 compared with control.

QPCR and Western blot were performed to analyze the mRNA and protein expression of TTF-1 in rat ATII cells. After 24 h of transfection, TTF-1 expression was markedly inhibited by shRNA-TTF-1 in rat ATII cells (Figure 9).

### 3.9. Liraglutide Promoted SP-A Expression by Increasing TTF-1 in Rat ATII Cells

**Figure 10:**
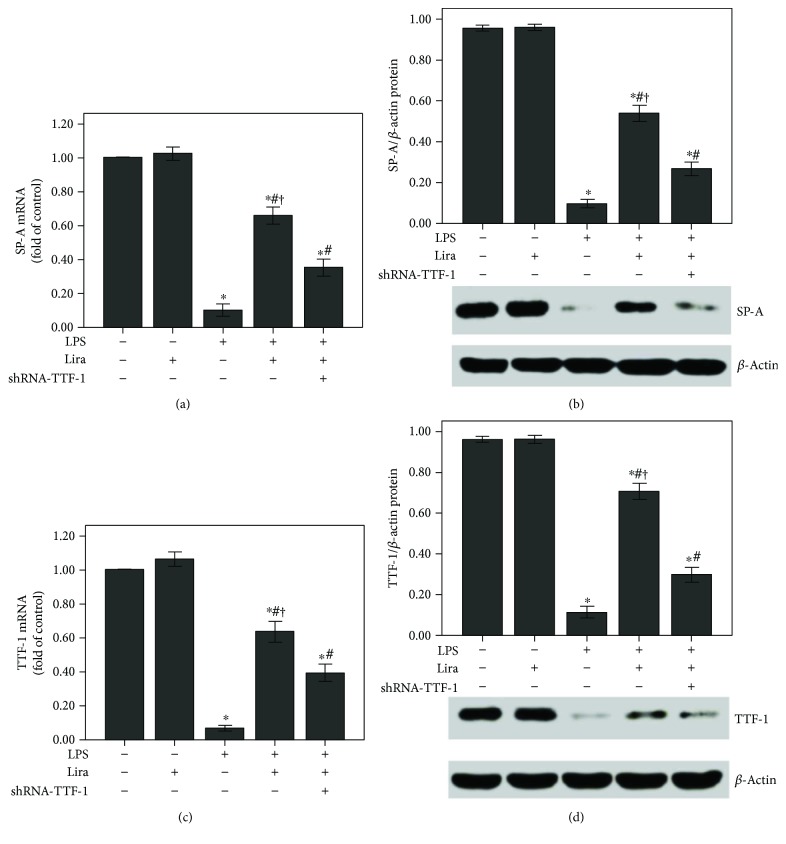
Liraglutide increased the expression of SP-A and TTF-1 in rat ATII cells. ShRNA-TTF-1-transfected and nontransfected ATII cells were pretreated with liraglutide (100 nM) for 4 h and then stimulated with LPS (100 ng/mL) for 4 h. (a and c) QPCR was used to analyze the mRNA expression of SP-A and TTF-1. (b and d) Western blot was performed to analyze the protein expression of SP-A and TTF-1. Quantitative data were presented as mean ± SEM (*n* = 5). ^∗^*P* < 0.05 compared with control. ^#^*P* < 0.05 compared with LPS. ^†^*P* < 0.05 compared with LPS+Lira+shRNA-TTF-1.

As shown in Figures 10(a) and 10(b), the expression of SP-A was notably reduced after 4 h of LPS stimulation. Liraglutide remarkably enhanced SP-A expression in ATII cells after LPS stimulation. Subsequently, LPS-induced suppression of TTF-1 expression was also notably abolished by liraglutide in ATII cells (Figures 10(c) and 10(d)). Furthermore, our data also demonstrated that these effects of liraglutide were substantially abrogated by shRNA-TTF-1.

## 4. Discussion

In the current study, our results showed that LPS-induced pulmonary inflammation, pulmonary edema, increase in insulin level and alveolar cell injuries, and LPS-induced reduction of SP-A expression were markedly compromised by GLP-1 analogue liraglutide both in vivo and in vitro. Furthermore, our data also indicated that this SP-A-enhancing property of liraglutide was most likely mediated via the TTF-1 signaling pathway.

ALI/ARDS, characterized by damage to the alveolar-capillary barrier, is induced by self-amplified and uncontrolled lung inflammation [4]. Although substantial progress has been made in the understanding of ALI/ARDS, effective treatments are still limited in clinical practice.

GLP-1 is mainly synthesized and secreted by intestinal L-cells [18]. GLP-1 analogues, including liraglutide and exenatide, have been successfully used in type 2 diabetes mellitus treatment. Several recent studies have confirmed that GLP-1R is also expressed in many extrapancreatic tissues, including endothelial cells, airway and alveolar epithelial cells, macrophages, gastrointestinal tract, myocardium, and kidney [15–17]. A previous study revealed the protective role of liraglutide in a murine model of obstructive lung disease [16]. Moreover, our previous investigation also showed that OVA-induced airway inflammation and mucus hypersecretion were markedly inhibited by liraglutide in mice [15]. Meanwhile, our other study demonstrated that bleomycin-induced pulmonary inflammation and fibrosis were notably attenuated by liraglutide in mice [22]. After 72 h of LPS injection, severe and typical pathological changes in the lungs were observed (Figure 2(a)). Our results showed that these typical pathological alterations and enhanced lung injury scores were both significantly improved by liraglutide (Figures 2(a) and 2(b)). Meanwhile, according to previous studies, MPO activity is a marker of accumulation and activation of neutrophils in inflammatory processes [6, 21]. Our findings demonstrated that LPS-induced increase in MPO activity in the lung was largely inhibited by liraglutide (Figure 2(d)). At the initial stage of infection-induced acute inflammation, pathogen-associated molecular patterns (PAMPs), including LPS and CpG DNA, are recognized and interacted by inflammatory cells with pattern recognition receptors (PPRs), such as mannose receptor (MR) and toll-like receptors (TLRs) [29, 30]. Following this, a wide range of inflammatory mediators, including TNF-*α*, IL-6, and IL-1*β*, are released [29, 30]. In our study, we found that LPS-induced increase in the number of total cells, neutrophils, and macrophages and LPS-induced increase in TNF-*α*, IL-6, and IL-1*β* levels in BALF were remarkably compromised by liraglutide (Figure 3). Additionally, noncardiogenic pulmonary edema is another major pathological feature of ARDS, a major cause of the failure of oxygenation [31]. Our data showed that LPS-induced increase in the W/D ratio was markedly reduced by liraglutide (Figure 2(c)). Therefore, these findings indicated that LPS-induced pulmonary inflammation, injury, and edema were substantially attenuated by liraglutide in mice.

GLP-1 is a key regulator of insulin secretion. It has been found that the effect of GLP-1 in promoting pancreatic *β* cell insulin secretion was dependent on blood glucose concentration [32]. Fransson et al. showed that liraglutide did not influence serum insulin level in nondisease mice; however, corticosterone-induced hyperinsulinemia was alleviated by liraglutide [33]. It is well known that insulin tolerance is impaired in severe conditions, such as ARDS, severe sepsis, and severe burn. Inflammation plays a critical role in insulin intolerance in these conditions. Landgraf et al. found that LPS-induced insulin tolerance impairment was alleviated by leptin, resulting from its anti-inflammatory effect, in mice [34]. In the current study, our data showed that LPS-increased fasting serum insulin was suppressed by liraglutide (Figure 7). Our data also demonstrated that fasting serum insulin level was not influenced by liraglutide in nondisease mice. This result indicates that LPS-increased insulin level was remarkably suppressed by liraglutide in mice, possibly stemming from its anti-inflammatory effect.

PS is a complex of various lipids (90%) and proteins (10%), mainly synthesized by ATII cells [35, 36]. PS is responsible for increasing pulmonary compliance, preventing the lung from collapsing at the end of expiration, maintaining fluid balance in the lung, regulating the size of alveoli, and modulating the lung's innate immune system [3, 35, 36]. Lamellar bodies, the main sites for the synthesis and metabolism of surfactants, are the specialized organelles of ATII cells [1, 36]. Simultaneously, it is well known that mitochondria, the powerhouse of the cell, is the most sensitive and vulnerable organelle, responding to injury and hypoxia. Even slight injury can cause mitochondrial dysfunction and structure changes which usually is the first and most common finding after cell damage. ATII cell is the major injury target of ARDS [21]. Some investigations have shown that the degree of functional impairment and structural abnormality of lamellar bodies and mitochondria were correlated with the severity of ALI [21, 37, 38]. Our data also showed that LPS-induced ultrastructural alterations, particularly lamellar bodies and mitochondria, in ATII cells were notably attenuated by liraglutide in mice (Figure 4). Thus, this result suggested the protective role of liraglutide in LPS-induced ATII cell injury.

SP-A is the most abundant protein component of PS [1, 39]. SP-A plays an indispensable role in tubular myelin formation and recycling of PS [35]. Tubular myelin, the precursor of a monomolecular surfactant form, is an ultrathin film lining the surface of the alveolus [35]. Moreover, a number of studies have reported that SP-A is essential for innate immunity in the lung [3, 10, 39]. Many previous studies have shown that SP-A expression is significantly reduced in ALI/ARDS [40, 41]. It is well known that reduction of PS is essential for decreased pulmonary compliance and edema in ALI/ARDS. There is broad evidence that the expressions of SP-A and other PS components were reduced after ATII injury in ALI [21, 42–45]. Then, in the current study, our results also indicate that the reduction of SP-A expression in the lung was correlated with the severity of lamellar body damage and ATII cell injury in ALI (Figures 4 and 5). Therefore, we presumed that upregulation of SP-A might benefit in treating ARDS. Simultaneously, GLP-1 played a critical role in regulation of SP-A and lung development. Previously, it was found that SP-A expression was upregulated and the lung size was increased by liraglutide in fetal and neonatal rats [17]. Furthermore, another study also showed that streptozotocin-induced reduction of SP-A expression was prevented by liraglutide through the TTF-1 signaling pathway in a rat model of diabetes [46]. In the current study, our data demonstrated that LPS-induced downregulation of SP-A expression could be abolished by liraglutide (Figure 5).

TTF-1 is considered to be a central regulator of SP-A transcription in ATII cells [12, 13]. Meanwhile, it is reported that TTF-1 also played a role in LPS and other inflammatory stimulator-induced ATII cell injuries [42, 45]. Das et al. figured out that TNF-*α* could directly inhibit TTF-1 expression by binding the TTF-1 proximal promoter in H441 and primary alveolar type II cells [42]. TNF-*α*, one of the most critical inflammatory mediators, has been implicated in the pathogenesis of ARDS and inhibits surfactant protein levels [21, 45]. The TTF-1-binding element (TBE) has been identified in the promoter region of *SP-A* [12]. Chen et al. revealed that SP-A expression in rat ATII cells could be upregulated by leptin via the TTF-1 signaling pathway under hypoxic conditions [13]. In the current study, our data indicated that liraglutide upregulated SP-A expression through the TTF-1 signaling pathway in LPS-induced ALI (Figures 6 and 10).

SP-A is essential for modulating lung inflammation [3, 39]. Some studies have determined the anti-inflammatory role of SP-A in different conditions [10, 11]. Du et al. found that pneumonia severity and intestinal injury were notably attenuated by SP-A in a murine model of *Staphylococcus aureus* pneumonia [47]. Minutti et al. demonstrated that SP-A attenuated IFN-*γ*/LPS-induced alveolar macrophage activation [48]. Another study also reported that TTF-1 played a role in the regulation of pulmonary inflammation [49]. In the current study, our data revealed that the effects of liraglutide on LPS-induced pulmonary inflammation, pulmonary edema, increase in insulin level, and ATII cell injury were significantly blunted by shRNA-TTF-1. Thus, this result suggested that the effects of liraglutide were induced by increasing TTF-1 expression. Nevertheless, the underlying mechanism of how liraglutide regulates TTF-1 expression in ATII cells in ALI is still unclear. However, several studies, including our previous study, found that the cAMP/PKA signal pathway was essential for the bioactivity of GLP-1 in different conditions [15, 18]. Steven et al. demonstrated that GLP-1 receptor activation in platelets by linagliptin and liraglutide attenuated LPS-induced microvascular thrombosis, systemic inflammation, vascular dysfunction, and end organ damage by a cAMP/PKA-dependent mechanism [18]. Therefore, we highly supposed that the cAMP/PKA signal pathway would play a critical role in liraglutide-induced TTF-1 expression in ATII cells in ALI. Meanwhile, we also supposed that one or more intermediate molecules probably were involved in this process. Thus, we presume that liraglutide-induced TTF-1 expression in ALI is indirect. And this is an important area that requires further research.

Additionally, it is reported that haploinsufficient mutations in *TTF-1* are associated with pulmonary disease in infants and with variable inhibitory effects on the expression of SPs in human [50, 51]. However, in our current study, shRNA-TTF-1 was given 3 days before LPS injection. The time period was too short to cause significant pulmonary histological changes. As shown in Figure 1, after 3 days of transfection, no pathological alterations were observed in the control, shRNA-scrambled, and shRNA-TTF-1 groups of mice. Meanwhile, in the current study, TTF-1 knockdown via shRNA is not sufficient to completely abrogate TTF-1 expression in ATII cells. TTF-1 conditional knockout mice should be used to further confirm this mechanism. Interestingly, we also noticed that liraglutide alone could not promote SP-A expression both in vivo and in vitro (Figures 5 and 10). This result can probably be attributed to the complex SP-A metabolic balance. The molecular mechanisms responsible for SP-A metabolic balance in ATII cells should be investigated.

For the safety and tolerance of liraglutide, a comparable high dosage of liraglutide (2 mg/kg) was given to mice in the current study [15, 22]. It is reported that body weight and food intake reduction are the major side effects of GLP-1 analogues in both human and animals [52, 53]. Then, we also observed that the food intake of mice with liraglutide administration was suppressed in our study. And the role of energy and food consumption in the pathogenesis of ALI needs to be elucidated in the future.

Taken together, our results indicated that liraglutide upregulated SP-A expression in ATII cells and attenuated pulmonary inflammation, pulmonary edema, and increase in insulin level in LPS-induced ALI, most likely through the TTF-1 signaling pathway, suggesting that liraglutide may be considered an effective drug for the potential treatment of ARDS in the future.
